# Gestation-specific reference intervals for comprehensive spot urinary steroid hormone metabolite analysis in normal singleton pregnancy and 6 weeks postpartum

**DOI:** 10.1186/s12958-015-0100-6

**Published:** 2015-09-04

**Authors:** Hiten D. Mistry, Nicole Eisele, Geneviève Escher, Bernhard Dick, Daniel Surbek, Christian Delles, Gemma Currie, Dietmar Schlembach, Markus G. Mohaupt, Carine Gennari-Moser

**Affiliations:** Department of Nephrology, Hypertension, Clinical Pharmacology and Clinical Research, University of Bern, 3010 Berne, Switzerland; Department of Obstetrics and Gynecology, University Hospital Bern, 3010 Berne, Switzerland; University of Glasgow, Institute of Cardiovascular and Medical Sciences, Glasgow, UK; Vivantes Clinic Berlin-Neukölln, Department of Obstetrics, Berlin, Germany; Division of Hypertension, Department of Nephrology, Hypertension, Clinical Pharmacology and Clinical Research, University of Bern, CH-3010 Berne, Switzerland

**Keywords:** Pregnancy reference values, Urine, Steroid metabolites

## Abstract

**Background:**

Normal pregnancy depends on pronounced adaptations in steroid hormone concentrations. Although in recent years, the understanding of these hormones in pregnancy has improved, the interpretation is hampered by insufficient reference values. Our aim was to establish gestation-specific reference intervals for spot urinary steroid hormone levels in normal singleton pregnancies and 6 weeks postpartum.

**Methods:**

Cross-sectional multicentre observational study. Women recruited between 2008 and 2013 at 3 University Hospitals in Switzerland (Bern), Scotland (Glasgow) and Austria (Graz). Spot urine was collected from healthy women undergoing a normal pregnancy (age, 16–45 years; mean, 31 years) attending routine antenatal clinics at gestation weeks 11, 20, and 28 and approximately 6 weeks postpartum. Urine steroid hormone levels were analysed using gas-chromatography mass spectrometry. Creatinine was also measured by routine analysis and used for normalisation.

**Results:**

From the results, a reference interval was calculated for each hormone metabolite at each trimester and 6 weeks postpartum. Changes in these concentrations between trimesters and postpartum were also observed for several steroid hormones and followed changes proposed for index steroid hormones.

**Conclusions:**

Normal gestation-specific reference values for spot urinary steroid hormones throughout pregnancy and early postpartum are now available to facilitate clinical management and research approaches to steroid hormone metabolism in pregnancy and the early postpartum period.

## Background

A successful healthy pregnancy is recognised by pregnancy-related changes in hormone concentrations, characterised by elevated levels of several circulating steroid hormones, which normally increase as pregnancy progresses [1, 2]. Changes in maternal hormone concentrations play a critical role in modulating the metabolic and immunological changes required for successful pregnancy outcome; they also have an important counterpart as the fetoplacental unit develops. There is interest in how endogenous steroid hormones and their respective metabolites have influence or are altered in studies during pregnancy with respect to fetal size, preterm birth, multiple pregnancies, regulation of partition, hypertensive disorders of pregnancy and other conditions [3–5]. Many of these previously published studies on steroid metabolite concentrations have produced inconsistent findings, mainly due to technological issues or lack of specificity. However, the development of sensitive mass spectrometry based assays that can accurately measure individual steroid hormone concentrations [6], allows improvement of our understanding of gestation-dependent trends of these hormones. As many conventional assays such as radioimmuno assays (RIA) or enzyme-linked immunosorbent assays (ELISA) are compromised by significant cross-reactivity and lack of specificity, future analyses will require mass spectrometry or other more specific techniques [7]. Earlier studies using GC-MS-based techniques in pregnancy reported analyses in different biological fluids, such as in plasma samples [8], in low numbers of pregnant women [9], in disease states [10, 11] and providing only partial steroid hormone panels [9].

Physiological changes in pregnancy, including 50 % plasma volume expansion or significant changes in binding protein concentrations, mean that plasma levels and non-pregnant urinary steroid hormone reference intervals may not be appropriate in pregnancy [12]. Although in recent years, our understanding of the role of these hormones in pregnancy has improved, the interpretation of these levels is still difficult. We are not aware of any previous work reporting spot urinary steroid metabolite profiles during pregnancy, calculating reference intervals recommended for cross-sectional data that varies with gestational age [13, 14].

Given the importance of changes in steroid hormones during pregnancy and that reliable, clinically and scientifically useful reference intervals are not available for urine; we set out to determine gestation-specific reference intervals in a cross-sectional study of women during normal pregnancy and 6 weeks postpartum. Furthermore, due to the problems associated with collection of 24 h urine, especially during pregnancy, an advantage of this study was to produce reference values using spot urines from 3 different populations.

## Materials and methods

### Subjects

A first set of cross-sectional healthy pregnant women recruited to the Bernese pregnancy registry were included in the study. Visits were at week 11 ± 2 (*n* = 25), 20 ± 2 (*n* = 32), 28 ± 2 (*n* = 26) and approximately 6 weeks postpartum (*n* = 40). The study was approved by the ethics committee of the Canton of Berne, Switzerland.

A second set of samples were derived from a large prospective pregnancy cohort from the Queen Mother’s, Princess Royal Maternity and Southern General Hospitals in Glasgow, Scotland. First trimester samples (11 ± 2 weeks’ gestation; *n* = 46) were taken at booking. The study was approved by the ethics committee of the West of Scotland Research Ethics Committee.

The third cohort of samples consisted of women from Graz, Austria. Cross-sectional samples were obtained from each trimester (weeks 11 ± 2 (*n* = 10), 20 ± 2 (*n* = 8) and 28 ± 2 (*n* = 14)) following ethical approval from the University of Graz, Austria.

All study subjects included from the 3 cohorts were in keeping with the declaration of Helsinki and all participants provided written, informed consent. Only those women maintaining a regular, normotensive, uncomplicated pregnancy without gestational diabetes, hepatic, renal or other obvious diseases with no fetal abnormalities such as fetal growth restriction were included in this study. Clinical data were prospectively collected and either existing hospital files or the woman’s personal history reviewed to exclude any pre-existing disease, including pregnancy outcome. In total, 81 samples were used from the first trimester and 40 for all other trimesters and postpartum.

### Urine sampling

Morning fasting urine samples were obtained from pregnant women as has been described for population-based assessments [15]. In all urine collections, sodium and creatinine concentrations were determined using routine methods. Urine aliquots were stored at −80 °C until further analysis.

### Assessment of urinary steroid hormones by GC-MS

Steroid hormone measurements for samples from all 3 cohorts were conducted in Bern using the same GC-MS method, as described earlier [6, 7, 16]. In short, preparation consisted of pre-extraction, enzymatic hydrolysis, extraction from the hydrolysis mixture, derivatisation and gel filtration. All solvents were purchased from Merck, USA). Medroxyprogesterone (2.5 μg; Steraloids, Inc. Switzerland) was added as recovery standard to 1.5 ml urine. The sample was extracted on a Sep Pak C18 column (Waters, Switzerland), dried, reconstituted in 0.1 M acetate buffer (pH 4.6) and hydrolyzed with powdered Helix pomatia enzyme (12.5 mg, Sigma-Aldrich, UK) and 12.5 μl of β-glucuronidase/arylsulfatase liquid enzyme (Roche, USA) at 55 °C for 3 h. The resulting free steroids were extracted on a Sep Pak C18 cartridge and 2.5 μg stigmasterol (Steraloids, Inc. Switzerland) plus 0.15 μg of 3β5β-TH-Aldo (custom-made by Taros Chemicals, Germany) were added as a standard for derivatisation and chromatography. The percentage recovery following extraction for all the assays was >90 %. The samples were derivatised to form methyloxime-trimethylsilyl ethers. The derivatives were purified by gel filtration on Lipidex 5000 (Perkin Elmer, USA) columns. Analysis was performed on a Hewlett-Packard gas chromatograph 6890 (Hewlett-Packard, Palo Alto, California, USA) with mass selective detector 5973 by selective ion monitoring (SIM). A steroid mixture containing a known amount of all steroid metabolites to be measured was analysed on a regular basis to act as a calibration standard. To control extraction variability, a control sample was run on each extraction panel. All steroid concentrations were normalised to urine creatinine values.

### Assessment of urinary creatinine concentrations

Urinary creatinine concentrations were measured either in the clinical laboratory or by a validated QuantiChromTM Creatinine Assay Kit (DICT-500; BioAssay Systems, Switzerland), with intra-assay coefficient of variations for both assays <5 %. Both assays were based on the same method and reference samples were analysed on both platforms to ensure similar results; measurement variability between the 2 techniques was less than 5 %.

### Statistics

Descriptive statistics, including upper and lower bounds of reference intervals were calculated for each metabolite separately by trimester and postpartum using SPSS version 22. Reference intervals are described as the 2.5 and 97.5th percentiles, centred around the median with their corresponding 90 % confidence intervals [17]. Virtanen *et al.*, states that sample sixes above 30 had smaller changes in SDs and CVs in reference intervals and thus at least 40 samples for each time point were used [14]. Data was found to be non-parametric following the Kolmogorov-Smirnov test for normality of data distribution, thus robust methods, with bootstrapping was used, which utilises an interactive process to estimate location and spread of the data [18].

## Results

### Participants

Basic demographic, obstetric and pregnancy description data of the women in this study are detailed in Table 1. Overall, the 3 cohorts were well matched with no significant differences observed for all demographic data. The majority of women who participated in this study were White European (>95 %) from all 3 centres.

**Table 1:** Basic demographic and pregnancy data from there cohorts used in the study

Parameter	Bern	Glasgow	Graz
Maternal Age (yrs)	31.5 ± 3.8	29.6 ± 6.3	31.8 ± 5.3
Booking BMI (kg/m^2^)	25.5 ± 3.8	24.3 ± 5.4	23.8 ± 3.7
Gestational age at delivery (Wks)	39 ± 1.2	40 ± 1.2	39 ± 1.3
Birthweight (g)	3505 ± 658	3477 ± 530	3304 ± 346
Baby gender (% male)	51	46	40

### Reference intervals

All steroid hormone concentrations, normalised to creatinine, grouped by trimester and steroid hormone are given in Tables 2, 3, 4 and 5 and the postpartum values are presented in Table 6, including the 2.5 and 97.5 % reference intervals. For ease of clinical use, the mean and median value for each steroid hormone metabolite has also been calculated, normalised to creatinine concentrations (Tables 2, 3, 4, 5 and 6).

**Table 2:** Steroid hormone metabolite[μg/mmol urinary creatinine]

	1^st^ trimester (*n* = 81)				2^nd^ trimester (*n* = 40)				3^rd^ trimester (*n* = 40)			
			Reference intervals				Reference intervals				Reference intervals	
move to a separate row	Mean ± SD	Median	2.5^th^ percentile [90 % CI]	97.5^th^ percentile [90 % CI]	Mean ± SD	Median	2.5^th^ percentile [90 % CI]	97.5^th^ percentile [90 % CI]	Mean ± SD	Median	2.5^th^ percentile [90 % CI]	97.5^th^ percentile [90 % CI]
Androsterone	322 ± 196	289	40.0 [36.0, 91.7]	764 [740, 780]	209 ± 172	159	17.3 [16.6, 55.5]	746 [665, 748]	237 ± 274	134	5.9 [5.5, 38.0]	1409 [844, 1423]
Etiocholanolone	231 ± 141	211	31.0 [30.9, 65.3]	617 [542, 688]	149 ± 95.4	126	24.0 [23.7, 61.5]	518 [355, 522]	163 ± 176	108	6.5 [6.0, 34.9]	774 [707, 776]
11-Oxo-Etiocholanolon	38.6 ± 18.7	37.6	6.3 [1.7, 11.5]	84.4 [74.1, 87.0]	38.0 ± 19.0	35.1	6.4 [6.3, 15.3]	77.8 [72.7, 77.9]	49.1 ± 52.3	36.7	5.8 [5.8, 13.5]	325 [125, 325]
11β-Hydroxy-Androsterone	89.8 ± 53.3	75.1	20.8 [17.1, 23.1]	233 [212, 234]	70.8 ± 44.3	62.6	7.3 [7.1, 21.2]	192 [188, 193]	94.3 ± 123	58.7	4.9 [4.5, 21.4]	716 [315, 726]
11β-Hydroxy-Etiocholanolon	31.5 ± 22.1	29.3	5.7 [4.8, 8.6]	83.8 [65.3, 163]	39.5 ± 28.6	28.6	3.2 [3.1, 11.6]	112 [97.7, 112]	43.4 ± 28.8	37.0	10.6 [10.5, 12.7]	141 [105, 141]
Dehydroepiandrosterone	86.2 ± 158	23.1	1.5 [1.5, 4.4]	759 [569, 774]	49.6 ± 87.5	18.4	0.7 [0.7, 2.1]	380 [348, 380]	61.6 ± 167	12.0	1.3 [1.3, 2.5]	919 [520, 929]
5-Androstene-3β,17β-diol	26.8 ± 25.6	18.8	1.4 [0.9, 4.1]	125 [78.0, 144]	20.1 ± 16.1	16.0	0.9 [0.9, 5.1]	85.3 [55.2, 86]	25.5 ± 29.8	17.9	1.0 [1.0, 5.5]	175 [69.0, 175]
5-Androstene-3β,17β-triol	17.3 ± 57.2	5.3	0.7 [0.7, 1.8]	225 [92.6, 454]	10.9 ± 33.0	2.6	0.8 [0.8, 1.0]	164 [139, 165]	9.9 ± 20.8	2.6	0.7 [0.7, 0.9]	115 [51.5, 115]
16α-Hydroxy-dehydroepiandrostrerone	96.4 ± 101	60.7	2.9 [1.2, 14.4]	415 [277, 600]	107 ± 107	88.7	2.1 [2.0, 12.5]	569 [259, 577]	154 ± 340	77.5	3.4 [3.3, 13.0]	2139 [376, 2139]
5-Androstene-3β,16α,17β-triol	84.3 ± 75.0	55.8	9.2 [7.5, 22.2]	278 [209, 306]	65.4 ± 60.0	52.8	11.7 [11.7, 27.7]	313 [127, 317]	107 ± 155	60.2	12.4 [12.3, 18.5]	868 [454, 868]
5-Pregnene-3β,16α,17,20α-triol	48.2 ± 56.5	36.8	1.2 [0.9, 3.5]	317 [161, 397]	20.6 ± 25.5	13.6	0.9 [0.9, 2.1]	151 [50.7, 154]	38.1 ± 111	11.4	1.7 [1.7, 2.9]	690 [107, 690]
Testosterone	2.0 ± 1.8	1.6	0.3 [0.2, 0.5]	9.4 [6, 11.1]	2.4 ± 3.2	1.3	0.3 [0.3, 0.4]	18.5 [7.4, 18.8]	3.6 ± 5.6	2.0	0.5 [0.5, 0.7]	27.8 [27.8, 28.1]
5α-Dihydrotestosterone	4.6 ± 3.2	3.6	0.6 [0.5, 1.1]	12.3 [11.4, 12.9]	3.7 ± 2.6	3.2	0.5 [0.5, 1.2]	11.1 [10.1, 11.1]	5.7 ± 3.7	4.6	0.4 [0.4, 1.7]	19.1 [11.3, 19.3]

**Table 3:** Steroid hormone metabolite[μg/mmol urinary creatinine]

	1^st^ trimester (*n* = 81)				2^nd^ trimester (*n* = 40)				3^rd^ trimester (*n* = 40)			
			Reference intervals				Reference intervals				Reference intervals	
move to a separate row	Mean ± SD	Median	2.5^th^ percentile [90 % CI]	97.5^th^ percentile [90 % CI]	Mean ± SD	Median	2.5^th^ percentile [90 % CI]	97.5^th^ percentile [90 % CI]	Mean ± SD	Median	2.5^th^ percentile [90 % CI]	97.5^th^ percentile [90 % CI]
Estriol	135 ± 151	79.9	10.6 [3.5, 17.0]	545 [489, 901]	975 ± 584	836	143 [143, 2881]	2877 [2660, 2881]	2651 ± 2651	1955	636 [636, 764]	15192 [9171, 15116]
17β-Estradiol	9.1 ± 6.7	7.3	0.7 [0.6, 2.1]	26.4 [24.8, 28.8]	16.6 ± 12.0	14.5	1.8 [1.8, 4.6]	63.5 [45.0, 64.0]	25.2 ± 25.2	24.0	2.3 [2.3, 8.9]	117 [111, 117]
Progesterone metabolites												
Pregnenolone	14.2 ± 10.0	12.5	2.8 [2.6, 4.2]	53.0 [41.3, 54]	18.0 ± 14.7	14.9	1.9 [1.9, 3.3]	65.3 [57.3, 65.5]	27.9 ± 42.4	16.9	4.2 [4.2, 6.2]	265 [94.2, 265]
17-Hydroxypregnanolone	91.0 ± 76.4	79.5	14.6 [9.1, 23.1]	229 [202, 599]	88.3 ± 55.5	72.8	18.8 [18.7, 41.9]	247 [238, 247]	189 ± 253	113	34.1 [34.2, 50.6]	1298 1051, 1298]
Pregnanediol	1269 ± 830	1110	215 [154, 416]	4257 [3093, 4405]	1765 ± 1291	1461	459 [457, 633]	7514 [4181, 7602]	3982 ± 3762	2877	785 [779, 1162]	21112 [11216, 21112]
Pregnanetriol	209 ± 114	191	53.9 [34.2, 80.9]	455 [378, 774]	182 ± 94.0	153	35.8 [34.6, 99.6]	480 [443, 481]	287 ± 321	191	53.9 [53.4, 89.2]	1865 [970, 1865]
11-Oxopregnanetriol	2.7 ± 2.7	2.0	0.4 [0.3, 0.7]	11.8 [10.5, 14.7]	2.8 ± 4.4	1.5	0.3 [0.3, 0.7]	26.4 [10.0, 26.9]	11.3 ± 29.2	1.9	0.2 [0.2, 0.5]	151 [99.2, 1501]

**Table 4:** Steroid hormone metabolite[μg/mmol urinary creatinine]

	1^st^ trimester (*n* = 81)				2^nd^ trimester (*n* = 40)				3^rd^ trimester (*n* = 40)			
			Reference intervals				Reference intervals				Reference intervals	
move to a separate row	Mean ± SD	Median	2.5^th^ percentile [90 % CI]	97.5^th^ percentile [90 % CI]	Mean ± SD	Median	2.5^th^ percentile [90 % CI]	97.5^th^ percentile [90 % CI]	Mean ± SD	Median	2.5^th^ percentile [90 % CI]	97.5^th^ percentile [90 % CI]
Tetrahydro-11-deoxycortisol	17.1 ± 10.1	15.5	2.7 [1.2, 6.9]	43.1 [35.1, 62.5]	23.2 ± 11.5	21.2	5.2 [5.1, 11.3]	56.2 [51.9, 56.3]	37.1 ± 27.4	31.6	8.9 [8.9, 11.4]	146 [110, 147]
Tetrahydro-11-deoxycorticosterone	12.5 ± 8.8	10.2	1.7 [0.9, 3.8]	40.1 [33.9, 46.6]	35.3 ± 102	16.1	4.1 [4.1, 5.9]	643 [466, 629]	588 ± 1681	31.6	6.1 [6.1, 14.7]	8789 [5075, 8875]
Corticosterone metabolites												
Tetrahydroaldosterone	15.4 ± 10.7	12.0	2.7 [2.1, 5.0]	49.8 [36.5, 65.6]	18.2 ± 19.5	11.4	1.6 [1.6, 3.9]	114 [40.6, 116]	22.9 ± 16.6	18.2	2.1 [2.1, 7.1]	87.1 [55.1, 87.9]
Tetrahydro-11-dehydrocorticosterone	33.6 ± 23.7	26.7	3.9 [3.2, 10.9]	108 [88.5, 130]	35.1 ± 19.3	30.3	10.2 [10.1, 15.8]	89.4 [87.1, 89.4]	88.0 ± 88.9	56.1	13.4 [13.3, 27.4]	385 [371, 386]
Tetrahydrocorticosterone	23.1 ± 12.7	21.4	3.4 [3.0, 8.1]	57.8 [48.5, 63.4]	25.8 ± 13.2	22.3	3.3 [3.2, 8.5]	57.9 [55.0, 58.0]	83.0 ± 107.3	43.4	15.2 [15.1, 22.5]	586 [344, 591]
5α-Tetrahydro-corticosterone	51.5 ± 39.3	39.8	5.9 [4.3, 13.9]	189 [143, 194]	33.8 ± 21.6	30.0	1.7 [1.6, 9.8]	93.0 [91.7, 93]	59.0 ± 62.4	33.8	5.6 [5.6, 15.0]	304 [241, 306]

**Table 5:** Steroid hormone metabolite[μg/mmol urinary creatinine]

	1^st^ trimester (*n* = 81)				2^nd^ trimester (*n* = 40)				3^rd^ trimester (*n* = 40)			
			Reference intervals				Reference intervals				Reference intervals	
move to a separate row	Mean ± SD	Median	2.5^th^ percentile [90 % CI]	97.5^th^ percentile [90 % CI]	Mean ± SD	Median	2.5^th^ percentile [90 % CI]	97.5^th^ percentile [90 % CI]	Mean ± SD	Median	2.5^th^ percentile [90 % CI]	97.5^th^ percentile [90 % CI]
Cortisone	34.6 ± 25.2	28.4	4.7 [4.4, 10.2]	110 [97.7, 113]	64.5 ± 39.8	53.3	14.1 [14.0, 25.8]	165 [161, 165]	79.1 ± 43.0	82.3	4.8 [4.7, 22.6]	180 [144, 181]
Tetrahydrocortisone	368 ± 165	349	77.6 [73.4, 162]	780 [644, 624]	308 ± 190	255	73.8 [73.4, 90.5]	948 [926, 947]	321 ± 210	267	5.9 [4.7, 101]	923 [748, 922]
α-Cortolon	227 ± 170	211	27.6 [17.7, 35.8]	715 [641, 725]	112 ± 138	75.9	15.7 [15.7, 26.0]	826 [583, 825]	148 ± 196	68.7	17.5 [17.5, 22.5]	752 [741, 752]
β-Cortolon	47.9 ± 37.9	43.4	2.5 [1.5, 5.1]	141 [122, 167]	27.5 ± 36.5	13.3	3.8 [3.8, 4.8]	179 [148, 178]	35.0 ± 55.1	11.2	3.6 [3.5, 7.5]	303 [122, 308]
Cortisone	34.6 ± 25.2	28.4	4.7 [4.4, 10.2]	110 [97.7, 113]	64.5 ± 39.8	53.3	14.1 [14.0, 25.8]	165 [161, 165]	79.1 ± 43.0	82.3	4.8 [4.7, 22.6]	180 [144, 181]
20α-Dihydrocortisone	5.4 ± 3.2	4.3	1.5 [1.4, 2.2]	14.9 [12.9, 15.2]	8.2 ± 3.8	7.2	2.4 [2.4, 4.2]	15.0 [14.3, 15.0]	9.8 ± 4.7	9.4	1.4 [1.4, 4.3]	19.1 [18.9, 19.1]
20β-Dihydrocortisone	22.2 ± 11.9	21.2	4.2 [3.7, 8.2]	52.7 [49.9, 54.2]	34.0 ± 16.5	35.3	5.7 [5.6, 12.6]	70.0 [67.4, 70.0]	45.8 ± 26.9	37.7	1.8 [1.7, 14.2]	109 [104, 109]
Cortisol metabolites												
Cortisol	29.1 ± 23.4	20	4.7 [1.5, 7.6]	102 [83.7, 119]	52.3 ± 42.3	38.8	7.9 [7.8, 17.3]	210 [168, 210]	98.2 ± 109	69.6	2.7 [2.5, 24.5]	641 [285, 650]
Tetrahydrocortisol	191 ± 99.6	178	26.2 [22.2, 73.1]	405 [389, 519]	136 ± 68.1	131	31.4 [31.2, 58.8]	296 [277, 296]	182 ± 202	123	15.7 [15.6, 52.9]	923 [823, 926]
5α-Tetrahydrocortisol	126 ± 111	90.0	13.2 [9.6, 27.8]	412 [379, 538]	54.7 ± 42.2	54.7	4.6 [4.6, 18.4]	158 [157, 158]	46.7 ± 55.2	29.2	1.3 [1.2, 10.3]	258 [218, 259]
α-Cortol	55.5 ± 32.0	49.9	8.9 [8.2, 18.5]	145 [111, 192]	37.4 ± 17.9	34.6	7.1 [6.9, 18.6]	107 [73.5, 108]	51.1 ± 56.5	35.1	6.0 [5.9, 12]	248 [235, 248]
β-Cortol	45.6 ± 22.8	39.9	14.9 [11.8, 21.0]	118 [10.0, 122]	32.4 ± 16.7	26.6	6.9 [6.7, 15.2]	80.0 [68.2, 80.3]	36.3 ± 33.5	24.1	6.3 [6.2, 13.2]	187 [101, 189]
20α-Dihydrocortisol	30.0 ± 33.7	19.7	1.9 [1.8, 6.6]	180 [117, 215]	60.4 ± 60.2	37.3	1.7 [1.6, 9.5]	258 [246, 258]	145 ± 203	74.8	5.1 [4.9, 18.3]	1049 [623, 1060]

**Table 6:** Postpartum urinary steroid hormone concentrations

	6 weeks postpartum (n = 40)			
Steroid μg per mmol creatinine			Reference intervals	
move to a separate row	Mean ± SD	Median	2.5^th^ percentile [90 % CI]	97.5^th^ percentile [90 % CI]
Androsterone	130 ± 116	99.8	6.5 [6.2, 24.2]	524 [482, 525]
Etiocholanolone	180 ± 120	149	19.4 [19.3, 42.7]	456 [423, 457]
11-Oxo-Etiocholanolone	51.0 ± 35.1	42.1	8.0 [8.0, 14.9]	156 [149, 156]
11β-Hydroxy-Androsterone	77.5 ± 51.4	71.1	4.4 [4.2, 19.1]	262 [149, 156]
11β-Hydroxy-Etiocholanolone	46.4 ± 36.6	38.3	4.8 [4.8, 9.3]	161 [150, 161]
Dehydroepiandrosterone	33.5 ± 57.1	10.4	0.8 [0.8, 2.7]	291 [160, 294]
5-Androstene-3β,17β-diol	14.2 ± 15.1	9.4	0.4 [0.4, 1.6]	68.0 [50.0, 68.4]
5-Androstene-3β,17β-triol	9.8 ± 21.5	3.1	0.2 [0.2, 0.6]	113 [76.5, 114]
16α-Hydroxy-dehydroepiandrostrerone	46.8 ± 86.8	22.8	0.9 [0.9, 3.1]	502 [212, 510]
5-Androstene-3β,16α,17β-triol	40.4 ± 45.9	29.6	3.7 [3.6, 9.5]	271 [116, 275]
5-Pregnene-3β,16α,17,20α-triol	13.5 ± 12.5	10.7	0.4 [0.4, 1.3]	59.5 [38.9, 60.0]
Testosterone	3.7 ± 5.7	1.4	0.1 [0.1, 0.5]	26.0 [22.9, 26.1]
5α-Dihydrotestosterone	2.5 ± 2.3	2.2	0.1 [0.1, 0.6]	12.4 [8.2, 12.3]
Oestrogen metabolites				
Estriol	119 ± 339	17.6	0.9 [0.9, 1.4]	2011 [557, 2049]
17β-Estradiol	2.9 ± 9.6	0.5	0.1 [0.05, 0.1]	58.7 [9.0, 60.0]
Progesterone precursor and metabolites				
Pregnenolone	9.8 ± 13.4	4.7	0.2 [0.2, 0.7]	52.5 [49.7, 52.2]
17-Hydroxypregnanolone	20.2 ± 41.4	9.9	0.9 [0.9, 2.4]	257 [76.6, 262]
Pregnanediol	257 ± 668	76.2	10.4 [10.3, 29.5]	4052 [1173, 4126]
Pregnanetriol	56.0 ± 49.7	44.3	5.9 [5.7, 20.8]	297 [144, 301]
11-Oxo-Pregnanetriol	2.8 ± 3.4	1.6	0.4 [0.4, 0.7]	17.7 [10.1, 17.8]
11-Deoxysteroid metabolites				
Tetrahydro-11-deoxycortisol	11.1 ± 8.6	9.8	0.7 [0.7, 3.0]	43.1 [30.6, 42.8]
Tetrahydro-11-deoxycorticosterone	28.2 ± 160	1.3	0.1 [0.1, 0.4]	989 [27.0, 1014]
Corticosterone metabolites				
Tetrahydroaldosterone	4.2 ± 4.6	2.6	0.5 [0.5, 1.1]	21.9 [19.3, 21.9]
Tetrahydro-11-dehydrocorticosterone	20.7 ± 23.5	14.6	0.4 [0.4, 3.3]	139 [53.9, 142]
Tetrahydrocorticosterone	23.8 ± 16.7	20.1	2.2 [2.2, 6.8]	71.3 [69.3, 71.3]
5a-Tetrahydrocorticosterone	35.7 ± 28.1	28.8	1.7 [1.6, 6.3]	143 [113, 143]
Cortisone metabolite				
Cortisone	28.2 ± 20.1	23.4	1.0 [1.0, 10.3]	101 [74.8, 101]
Tetrahydrocortisone	397 ± 233	373	58.8 [58.2, 120]	1034 [906, 1037]
α-Cortolon	122 ± 117	90.5	18.1 [18, 33.5]	707 [286, 718]
β-Cortolon	30.6 ± 40.4	6.1	0.2 [0.2, 1.5]	156 [141, 155]
20α-Dihydrocortisone	3.4 ± 2.7	2.7	0.2 [0.2, 1.1]	13.5 [9.7, 13.6]
20β-Dihydrocortisone	9.2 ± 6.9	8.1	0.5 [0.4. 3.1]	34.3 [24.5, 34.5]
Cortisol metabolites				
Cortisol	20.8 ± 19.1	13.8	0.9 [0.8, 4.1]	99.7 [66.7, 99.7]
Tetrahydrocortisol	256 ± 153	243	30.7 [30.3, 80.3]	723 [599, 726]
5α-Tetrahydrocortisol	97.1 ± 81.2	78.3	5.8 [5.4, 22.7]	381 [302, 383]
α-Cortol	41.1 ± 32.6	35.5	3.9 [3.8, 17.7]	203 [98.2, 206]
β-Cortol	55.3 ± 29.2	50.1	10.0 [9.9, 25.8]	130 [114, 130]
20α-Dihydrocortisol	17.3 ± 22.6	9.3	0.2 [0.2, 3]	127 [50.7, 127]
6β-Hydroxycortisol	149 ± 25.8	26.5	0.9 [0.9, 8.1]	149 [77.9, 149]
18-Hydroxycortisol	322 ± 55.1	41.7	1.5 [1.4, 4.9]	322 [130, 322]

### Androgen metabolites

**Fig. 1:**
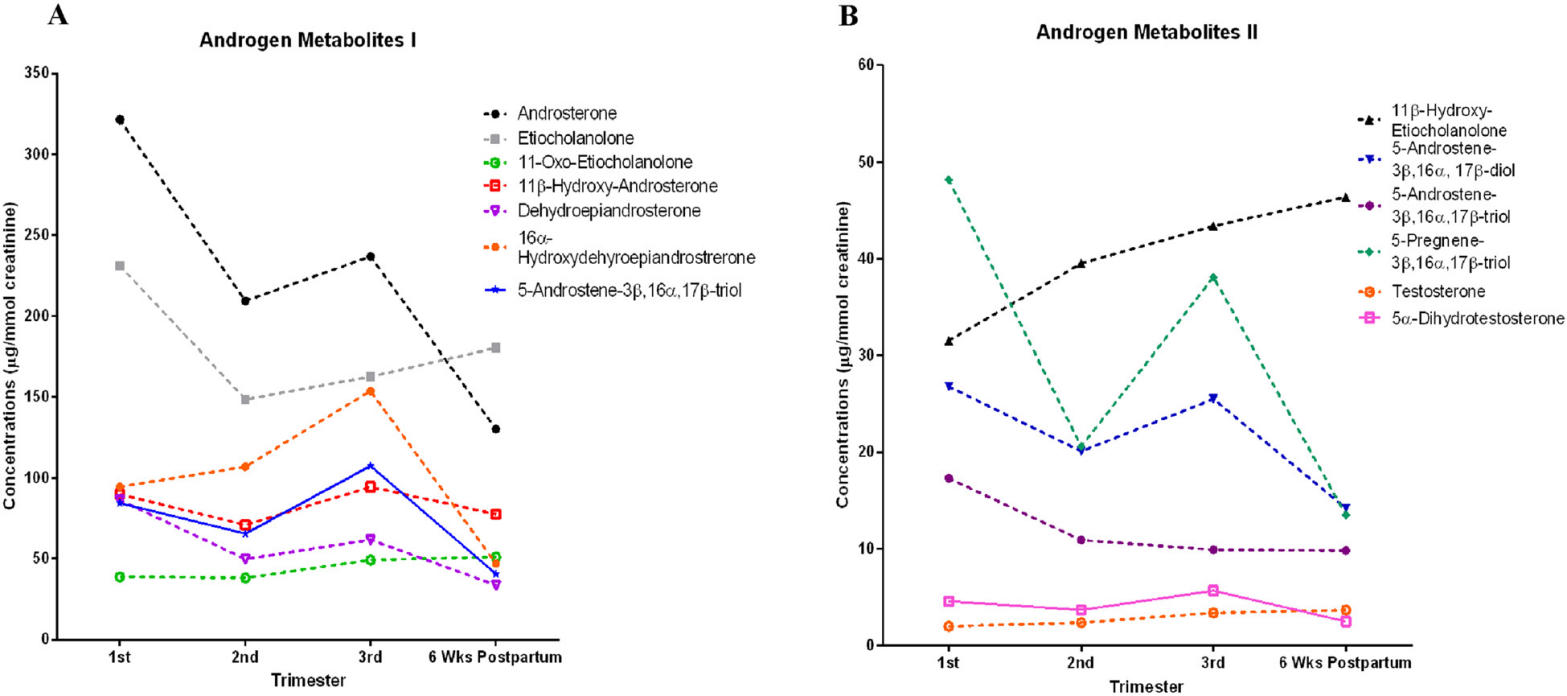
**a** and **b** Urinary concentrations of androgen metabolites in first, second, third trimester and six weeks postpartum. Each point is presented as mean corrected for creatinine concentrations (μg/mmol creatinine)

For clarity, androgens are presented in 2 graphs as follows: Fig. 1a: androsterone, etiocholanolone, 11-oxo-etiocholanolone 11β-hydroxy-androsterone, dehydroepiandrosterone, 16α-hydroxydehydroepiandrosterone and 5-androstene-3β,16α,17β-triol. Figure 1b: 11β-hydroxy-etiocholanolone, 5-androstene-3β,16α,17β-diol, 5-androstene-3β,16α,17β-triol, 5-pregnene-3β,16α,17,20α-triol, testosterone and 5α-dihydrotestosterone.

### Oestrogen metabolites

**Fig. 2:**
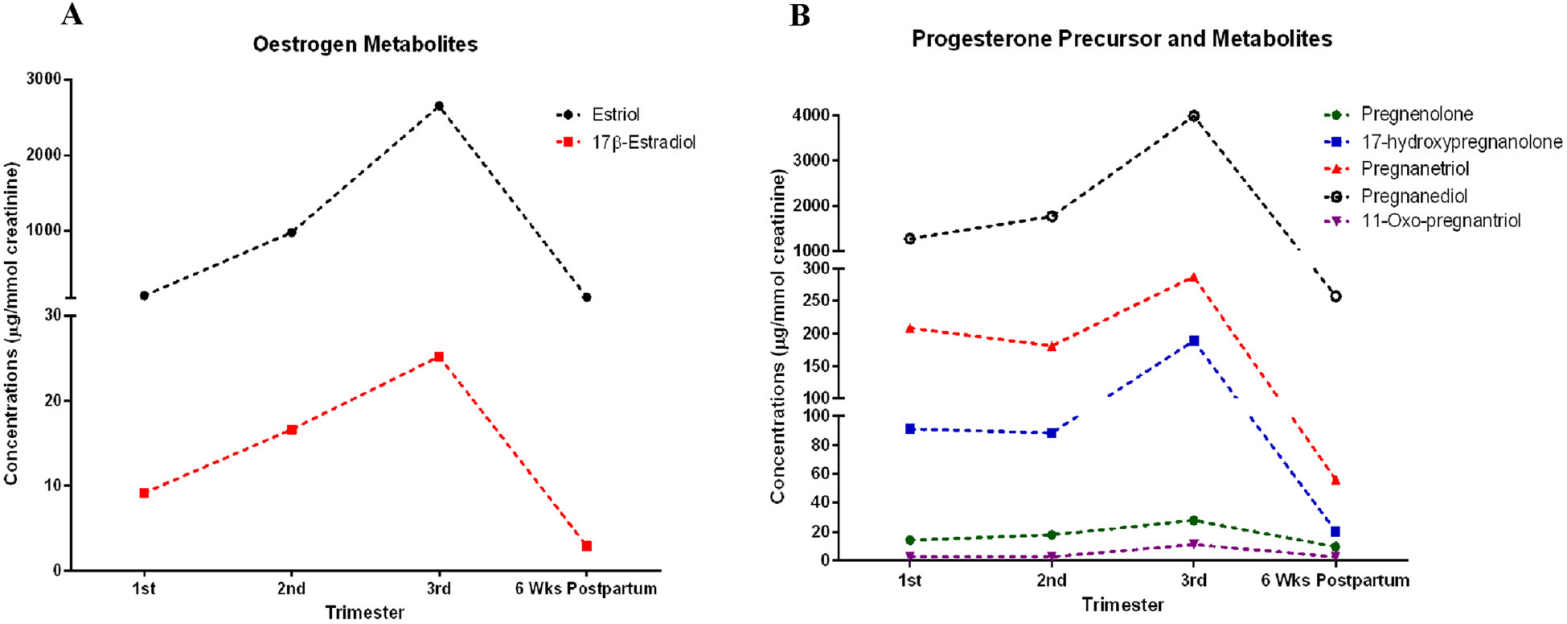
Urinary concentrations of (**a**) oestrogen metabolites and (**b**) progesterone precursor (pregnenolone) and progesterone metabolites in first, second, third trimester and six weeks postpartum. Each point is presented as mean corrected for creatinine concentrations (μg/mmol creatinine)

Both estriol and 17β-estradiol differed between the trimesters and postpartum (Fig. 2a); with the highest concentrations in the third trimester, followed by a dramatic decrease postpartum, lower than first trimester concentrations (Fig. 1a).

### Progesterone precursor and metabolites

The progesterone percursor, pregnenolone and all the progesterone metabolites (17-hydroxypregnanolone, pregnanetriol, pregnanediol, and 11-oxo-pregnanetriol) are presented in Fig. 2b. All metabolites increased between the 2^nd^ and 3^rd^ trimester, then subsequently fell postpartum.

### 11-deoxysteroid hormone metabolites

**Fig. 3:**
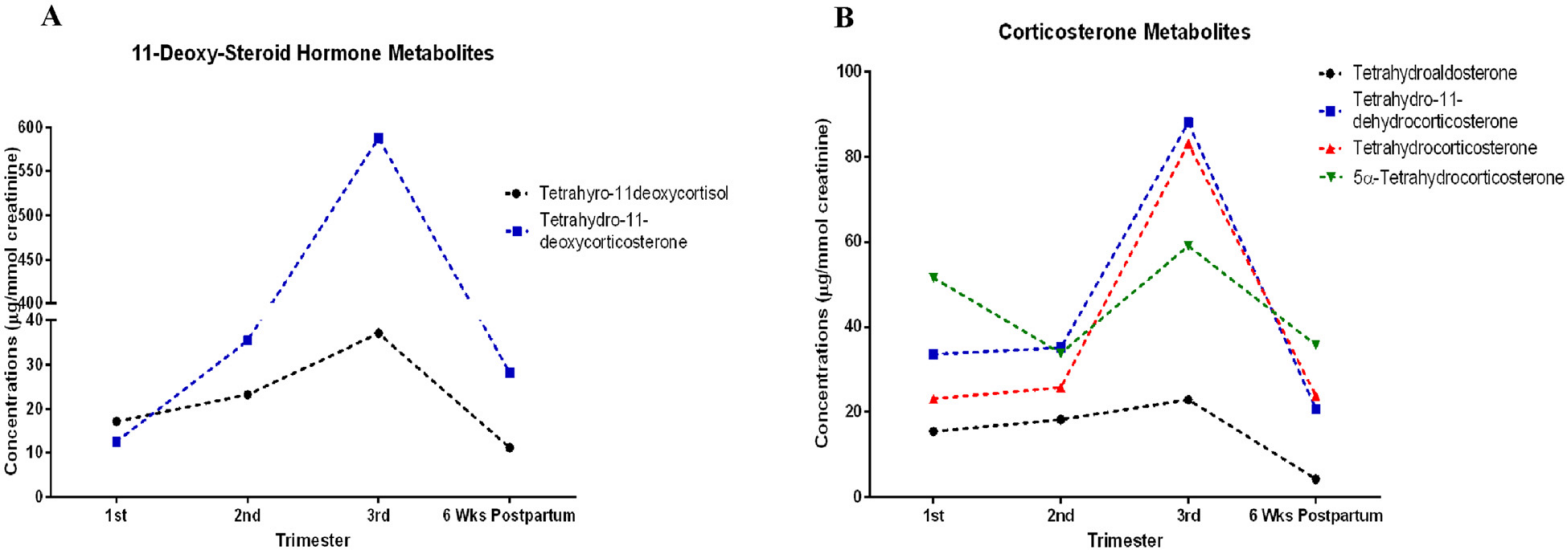
Urinary concentrations of (**a**) 11-Deoxy-steroid hormone metabolites and (**b**) corticosterone metabolites in first, second, third trimester and six weeks postpartum. Each point is presented as mean corrected for creatinine concentrations (μg/mmol creatinine)

Both tetrahydro-11-deoxycortisol and tetrahydro-11-deoxycorticosterone concentrations were increased with gestational age and decreased postpartum (Fig. 3a).

### Corticosterone metabolites

All metabolites differed between trimesters (Fig. 3b). Tetrahydroaldosterone and tetrahydrocorticosterone already increased throughout pregnancy and then subsequently decreased 6-weeks postpartum (Fig. 3b).

### Cortisol and cortisone metabolites

**Fig. 4:**
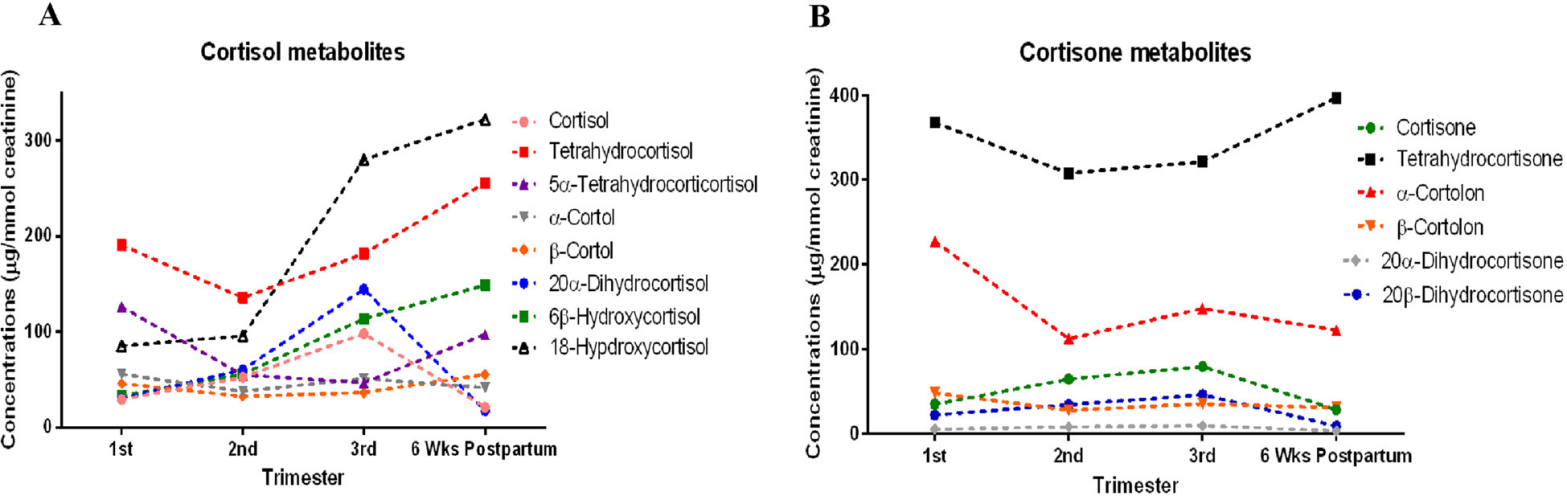
Urinary concentrations (**a**) cortisol metabolites and (**b**) cortisone metabolites in first, second, third trimester and six weeks postpartum. Each point is presented as mean corrected for creatinine concentrations (μg/mmol creatinine)

For clarity, cortisol and cortisone metabolites are presented in 2 graphs as follows: Fig. 4a: cortisol, tetrahydrocortisol, 5α-tetrahydrocortisol, α-cortol, β-cortol, 20α-dihydrocortisol, 6β-hydroxycortisol and 18-hydroxycortisol. Figure 4b: cortisone, tetrahydrocortisone, α-cortolon, β-cortolon, 20α-dihydrocortisone and 20β-dihydrocortisone. The most dramatic change was the increase in 18-hydroxycortisol during the 3 trimesters and postpartum.

For all steroid hormone metabolites measured, we tested whether any fetal gender specific differences were detectable. No significant differences (*P* < 0.05) were observed for any steroid hormone metabolites.

## Discussion

We have investigated the changes in spot urinary concentrations of the main steroid hormone metabolites throughout normal pregnancy and 6 weeks postpartum using a validated GC-MS method. Furthermore, we have used appropriate analysis techniques to calculate clinically relevant gestation-specific reference intervals. These data illustrate the profound changes in maternal steroidogenesis during pregnancy and indicate normal functional changes such as of the hypothalamic-pituitary-gonadal axis during pregnancy. A relationship between pregnancy outcome and steroid hormone availability such as for aldosterone has been established in the past by our group for aldosterone and cortisol [19, 20]. It have been known for many years that during the first 9 weeks of pregnancy, the corpus luteum and, the maternal ovary and adrenal cortex contribute to circulating concentrations of maternal hormones [21, 22]. After this time, the placenta becomes the predominant source of many maternal steroids [23].

These can then be used to improve management of steroid hormone related diseases in pregnancy, as well as encourage the appreciation of the changes that occur in steroid hormones during pregnancy. It will also allow clinicians to use gestation-specific reference ranges for management. Several steroid hormone metabolites are often combined to provide the clinician with a general picture of the individual’s steroid hormone production. This is useful as first point of investigation to obtain an integrated picture of the patient, to aid the identification of inherited metabolic disorders of steroid metabolism, and to perform non-invasive diagnostics. If normal values for pregnancy are established, even prenatal diagnosis of fetal disease might be feasible. Given the altered steroid hormone profile in pregnancy, these normal values might also allow discrimination between acquired, such as adrenal adenomas, and inborn errors of metabolism [7] (detailed on Table 7). Furthermore, some metabolites are still the subject of research investigations in the future. This could contribute to our knowledge of developmental changes in synthesis and metabolism of steroid hormones, such as the early diagnosis of congenital adrenal hyperplasia [24].

**Table 7:** Assignment of steroid hormone metabolites to their main reference

Full trivial name	Reference	Analyzed enzyme system
Androgen metabolites		
Androsterone	Androstenedione, testosterone, 5α-dihydrotestosterone, dehydroepiandrosterone	CYP17A1, POR, SRD5A2, HSD17B3
Etiocholanolone	Testosterone, dehydroepiandrosterone	CYP17A1, POR, SRD5A2, HSD17B3
11β-Hydroxy-androsterone	11β-hydroxy-androstenedione, cortisol	
Dehydroepiandrosterone	Dehydroepiandrosterone	HSD3B2
5-Androstene-3β,17β-diol	Testosterone, dehydroepiandrosterone	
5-Androstene-3β,17β-triol	Testosterone, dehydroepiandrosterone	
16α-Hydroxy-dehydroepiandrosterone	Dehydroepiandrosterone	
5-Androstene-3β,16α,17β-triol	Testosterone, dehydroepiandrosterone	
11β-Hydroxy-etiocholanolone	Testosterone, dehydroepiandrosterone, cortisol	
11-Oxo-etiocholanolone	Testosterone, dehydroepiandrosterone, cortisol	
Testosterone	Testosterone	
5α-Dihydrotestosterone	Testosterone	
Oestrogen metabolites		
Estriol	Estradiol	
17β-Estradiol	Estradiol	
Progesterone metabolites		
Pregnenolone	Pregnenolone	
17-Hydroxypregnanolone	17-Hydroxy-progesterone	CYP17A1, POR
Pregnanediol	Pregnenolone	POR
Pregnanetriol	17-Hydroxy-progesterone	CYP21A2, POR
11-Oxopregnanetriol	17-Hydroxy-progesterone	CYP21A2
5-Pregnene-3β,16α,17,20α-triol	17-Hydroxy-pregnenolone	HSD3B2
11-Deoxysteroid hormone metabolites		
Tetrahydro-11-deoxycortisol	11-Deoxycortisol	CYP11B1
Tetrahydro-11-deoxycorticosterone	Corticosterone	
Corticosterone metabolites		
Tetrahydrocorticosterone	11-Deoxycorticosterone	CYP17A1, POR, SRD5A2
5α-Tetrahydrocorticosterone	11-Deoxycorticosterone	CYP17A1, POR, SRD5A2
Tetrahydro-11-dehydrocorticosterone	Corticosterone	CYP17A1, POR
Tetrahydroaldosterone	Aldosterone	
Cortisol metabolites		
Cortisol	Cortisol	HSD11B2
Tetrahydrocortisol	Cortisol	CYP21A2, CYP11B1, CYP17A1, HSD3B2, POR, HSD11B2, H6PDH, HSD17B3
5α-Tetrahydrocortisol	Cortisol	CYP21A2, CYP11B1, CYP17A1, HSD3B2, POR, HSD11B2, H6PDH, HSD17B3, SRD5A2
α-Cortol	Cortisol	HSD11B2
β-Cortol	Cortisol	HSD11B2
20α-Dihydrocortisol	Cortisol	
6β-Hydroxycortisol	Cortisol	
18-Hydroxycortisol	Cortisol	
Cortisone metabolites		
Cortisone	Cortisol, cortisone	HSD11B2
Tetrahydrocortisone	Cortisol, cortisone	CYP21A2, CYP11B1, CYP17A1, HSD3B2, POR, HSD11B2, H6PDH, HSD17B3
α-Cortolone	Cortisol, cortisone	HSD11B2
β-Cortolone	Cortisol, cortisone	HSD11B2
20α-Dihydrocortisone	Cortisol, cortisone	
20β-Dihydrocortisone	Cortisol, cortisone	

The placenta is a site for active steroidogenesis, which depends on highly integrated and active interactions with both mother and fetus. As is well established, progesterone levels are important for normal feto-placental function, playing essential roles in controlling and maintaining the course of normal pregnancy [25, 26]. The rises in these metabolites during pregnancy and subsequent fall postpartum are thus the normal responses previously described [5]. Equally many of the changes in mineralocorticoids and in glucocorticoids also reflect the normal physiological responses to pregnancy and in preparation for parturition [27].

Androgens derived from the maternal adrenal tissue can undergo aromatization to form oestrogens [28]. Although clinical manifestations of androgen excess are uncommon in pregnant women, any physical changes in these metabolites from the normal changes described should be of concern. After the first 5 to 6 weeks of pregnancy, the major source of estradiol is the placenta, where enzymes convert maternal and fetal dehydroepiandrosterone to estradiol, estrone and estriol [22]. Near term, 50 % of estradiol synthesised in the placenta is derived from precursors in the fetal circulation; the remainder coming from the maternal circulations. This explains the increased levels of this and 17β-estradiol in the third trimester. It is well known that the expulsion of the placenta causes a decline in steroid levels [23], reflected and confirmed in the postpartum concentrations in the current study.

These normal values reflect known changes in index steroid hormone levels throughout pregnancy (e.g. increases in oestrogen metabolites in the third trimester; Fig. 2a). However, numerous studies might have benefited from the addition of MS-based to conventional RIA- or ELISA-based methods to obtain more accurate levels, by avoiding interference with steroid hormone binding, not missing important metabolites or simply by circumventing cross-reactivity. Furthermore, urinary measurements are not just a momentary picture, but integrate hormone production over a given time span. The high accuracy and specificity will now allow comprehensive clinical and scientific analysis in pregnant women, which has recently been postulated in the endocrine community [29]. The multi-centric setup avoids centre bias, although given the uncomplicated pre-analytics in steroid hormone metabolite analytics; such an issue is quite unlikely. Further advantages of urinary samples include less invasiveness, which is a huge benefit in pediatric and pregnant populations. We also expect that fetal pathologies might be identified by maternal urine sampling in pregnancy. Moreover, blood sampling reflects just a single time point whereas the urine sampling allows a cumulative integrated view of the steroid hormone metabolome over the sampling period. Upon calculation of specific metabolite ratios, conditions of adrenal origin and other disorders might be diagnosed without the need of timed sample collection [7]. A limitation of all steroid studies, including ours, is the high variation between patients, possibly due to the use of spot, as opposed to 24 h urine collections, and the variable physical activity of participating individuals. Conversely, this is also a strength of the study and obtaining accurate 24 h urine collections are problematic, particularly during pregnancy and thus spot urine offers a suitable and more practical method for assessment of steroid metabolites. In addition, a large degree of day-to-day variation within individuals has to be considered [30–33]. This study was conducted primarily in a population of white European descent and while the absolute values might be limited in their generality, the hormonal patterns reflecting the HPG and HPA axes might show sufficient similarities even in different genetic backgrounds [31, 33–35]. The addition of samples from 3 different populations, account for variations in diet and salt intakes and thus provides more accurate reference intervals. Future work will establish possible changes in these steroid profiles in 24 h urine collections and in pathological pregnancies.

## Conclusions

In summary, we have established, as far as we are aware, for the first time, gestation-specific GC-MS based reference values for spot urinary endogenous hormone metabolites during pregnancy, which may also be of interest in studies of maternal and fetal health and disease. We have created an easy to use functionally grouped trimester-based table (Tables 2, 3, 4, 5 and 6) for each hormone, which will be of use to those managing and studying women with respect to steroid hormone metabolites in pregnancy.
